# Rhodopsin gene copies in Japanese eel originated in a teleost-specific genome duplication

**DOI:** 10.1186/s40851-017-0079-2

**Published:** 2017-10-17

**Authors:** Yoji Nakamura, Motoshige Yasuike, Miyuki Mekuchi, Yuki Iwasaki, Nobuhiko Ojima, Atushi Fujiwara, Seinen Chow, Kenji Saitoh

**Affiliations:** 1 Research Center for Bioinformatics and Biosciences, National Research Institute of Fisheries Science, Japan Fisheries Research and Education Agency, 2-12-4 Fukuura, Kanazawa, Yokohama, Kanagawa 236-8648 Japan; 2 Present address: National Institute of Genetics, 1111 Yata, Mishima, Shizuoka, 411-8540 Japan; 3 Present address: Japan Fisheries Research and Education Agency, 2-3-3 Minatomirai, Nishi, Yokohama, Kanagawa 220-6115 Japan; 4 Present address: Tohoku National Fisheries Research Institute, Japan Fisheries Research and Education Agency, 3-27-5 Shinhama, Shiogama, Miyagi 985-0001 Japan

**Keywords:** Whole genome duplication, Teleostei, *Anguilla*, Rhodopsin paralogs, Visual adaptation, Phylogenomics, Synteny, Gene loss

## Abstract

**Background:**

Gene duplication is considered important to increasing the genetic diversity in animals. In fish, visual pigment genes are often independently duplicated, and the evolutionary significance of such duplications has long been of interest. Eels have two rhodopsin genes (*rho*), one of which (freshwater type, *fw-rho*) functions in freshwater and the other (deep-sea type, *ds-rho*) in marine environments. Hence, switching of *rho* expression in retinal cells is tightly linked with eels’ unique life cycle, in which they migrate from rivers or lakes to the sea. These *rho* genes are apparently paralogous, but the timing of their duplication is unclear due to the deep-branching phylogeny. The aim of the present study is to elucidate the evolutionary origin of the two *rho* copies in eels using comparative genomics methods.

**Results:**

In the present study, we sequenced the genome of Japanese eel *Anguilla japonica* and reconstructed two regions containing *rho* by de novo assembly. We found a single corresponding region in a non-teleostean primitive ray-finned fish (spotted gar) and two regions in a primitive teleost (Asian arowana). The order of *ds-rho* and the neighboring genes was highly conserved among the three species. With respect to *fw-rho*, which was lost in Asian arowana, the neighboring genes were also syntenic between Japanese eel and Asian arowana. In particular, the pattern of gene losses in *ds-rho* and *fw-rho* regions was the same as that in Asian arowana, and no discrepancy was found in any of the teleost genomes examined. Phylogenetic analysis supports mutual monophyly of these two teleostean synteny groups, which correspond to the *ds-rho* and *fw-rho* regions.

**Conclusions:**

Syntenic and phylogenetic analyses suggest that the duplication of rhodopsin gene in Japanese eel predated the divergence of eel (Elopomorpha) and arowana (Osteoglossomorpha). Thus, based on the principle of parsimony, it is most likely that the rhodopsin paralogs were generated through a whole genome duplication in the ancestor of teleosts, and have remained till the present in eels with distinct functional roles. Our result indicates, for the first time, that teleost-specific genome duplication may have contributed to a gene innovation involved in eel-specific migratory life cycle.

**Electronic supplementary material:**

The online version of this article (10.1186/s40851-017-0079-2) contains supplementary material, which is available to authorized users.

## Background

In the molecular evolution of organisms, gene duplication plays a pivotal role in preparing raw materials for evolution [1, 2]. Whereas mutations in single-copy genes are under strong negative selection pressure, redundant gene copies enable a wider spectrum of mutations mostly deleterious. Moreover, some mutations may contribute to sub- or neo-functionalization of copies, making creative evolutionary changes. Gene duplication can occur at the single gene, segmental or chromosomal level, and even whole genome duplications are possible, which may cause the largest scale of divergence of gene functions. In particular, two rounds of whole genome duplication events at initial stages of vertebrate evolution have been important topics for several decades [2–5]. In fish, the ancestor of teleosts (infraclass Teleostei) underwent an additional round of genome duplication (TSD; teleost-specific genome duplication) around 300 million years ago [6–8], which has also attracted the attention of evolutionary biologists. About 48% of extant vertebrate species are teleosts [9, 10], and these live in highly diverse aquatic environments; from tropical coral reefs or rain forests to limit-cooled water under ice in polar regions or highland glacial lakes, from shallow estuaries to abyssal ocean trenches or far offshore open ocean surface, from highly alkalic to acidic waters, and from open waters under the sun to deep into ever dark caves. Thus, the correlation of whole genome duplication and eco-physiological diversification in the teleost lineage has been a topic that has attracted both interest and controversy in evolutionary biology [11–13].

Sensory organs are indispensable for most organisms, of which vision is specifically important for agile animals such as fish. Because of the differential penetration of lights of different wave lengths, diverse light sensitivity may be observed among fish species inhabiting different environments [14]. Vision also physiologically and ontogenetically changes: for example, fish visual sensitivity changes according to habitats shift from freshwater to marine environments [15, 16] and to food shift from carotenoid rich crustacean plankton to blue-green colored pelagic fish [17, 18]. Diversification in light sensitivity at various wave lengths is achieved by evolutionary tuning and differential expression of visual pigment genes. Visual pigment genes have been subjected to studies of gene duplication. In general, fish species have five types of visual pigment genes, which enable effective perception of colors (ultraviolet, blue, green, and red) and dim-light. These subtypes are considered to have arisen in two rounds of whole genome duplications in the ancestor of vertebrates [19]. Regarding TSD, however, the signature remains obscure; one of the two duplicate copies of visual pigment genes that emerged as the result of TSD has been lost in most teleosts. Rather, within each of the five types, the visual pigment gene is often duplicated at the single gene level independently of the teleost lineages [20, 21]. Rhodopsin is the visual pigment protein working for dim light vision [22], and the gene structure has been determined in many animals. The encoding gene (*rho*) is universally intronless in teleosts, which is believed to be due to a reverse-transcriptional insertion of the spliced messenger RNA transcribed at a distant locus early in the ray-finned fish lineage [23]. The original gene with introns before the insertion is utilized as retinal rhodopsin in tetrapods, but as exorhodopsin in fish [24]. It has been reported that *rho* is retained in the single copy state in most teleosts [25], but a few exceptional species with duplicate *rho* are scattered among teleostean tree of life, such as zebrafish, pearl eye, conger, and eel [26–29].

**Fig. 1:**
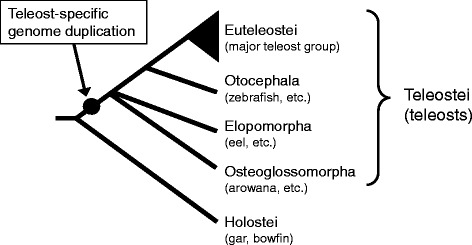
Phylogenetic relationship among the fish lineages examined in this study The timing of teleost-specific genome duplication (TSD) is shown by a closed dot.

Freshwater eels of the genus *Anguilla* are among the few catadromous migrating teleost fish, which grow in freshwater areas and descend to the sea for reproduction. Majority of teleostean species live in either marine (55.5%) or freshwater (42.7%) environments, and the other small fraction (1.8%) makes a round trip between the sea and freshwaters for growth and reproduction (diadromy) [30]. Catadromous fish comprise a further small fraction of diadromous fish. The majority of diadromous fish, such as salmons and freshwater gobies, spawn in freshwaters and grow in either marine or freshwater habitats (anadromous or amphidromous). The spawning area of Japanese eel *Anguilla japonica* Temminck & Schlegel, 1846 has been determined to be located at the southern part of the West Mariana Ridge [31, 32]. After years of living in the freshwater or near-shore habitats in the northeast Asia, Japanese eels metamorphose into silver stage at onset of early maturation and start oceanic migration to reach the above-mentioned open ocean spawning area. Biologging and tracking experiments have revealed that the freshwater eels perform diel vertical migration between upper and lower mesopelagic zones (c.a. 200–1000 m) during oceanic migration (see [33], and references therein). The life cycle of the freshwater eels is thus distinct from other teleosts, which might be associated with their phylogenetic position: the superorder Elopomorpha, as well as Osteoglossomorpha, is considered to have diverged from basal teleosts (Fig. 1) [34, 35].

Ambient light condition of the migrating eels should then change according to their life stages. Carlisle and Denton [16] reported that the wavelength of maximum absorption in visual pigments was different between eels caught in the river and the sea, and further studies have shown that this trait is due to the expression switching of two *rho* genes in retinal cells [29, 36–38]. Origin of these *rho*, namely *fw-rho* for freshwater and *ds-rho* for deep-sea in this study, is clearly the result of a gene duplication event [29], but when the event occurred remains unsolved. From the deep-branching phylogeny of rhodopsin and related genes in fish, the duplicate genes might be derivatives of TSD, but previous studies have not yielded a clear conclusion [19, 39, 40]. The timing of TSD is estimated to predate the occurrences of Elopomorpha and Osteoglossomorpha, and postdate the divergence between Teleostei and Holostei (gar and bowfin) (Fig. 1). Actually, spotted gar, *Lepisosteus oculatus*, possesses a single copy of intronless *rho* in a genomic region on linkage group 5 (LG5), which suggests that this species may have retained the ancestral structure of *rho* region before TSD [19]. With reference to Osteoglossomorpha, the genome of Asian arowana, *Scleropages formosus*, was recently sequenced [41], but the structure of its *rho* region has not been fully investigated.

In this study, we compared eel’s rhodopsin paralogs with those of other teleosts and vertebrates, such as Asian arowana and spotted gar, at the genomic level including synteny organization. The relationship between the evolutionary pathway of rhodopsin genes and migratory ecology is discussed.

## Methods

### Genome sequencing and assembly

All experiments were conducted following principles and procedures approved by the guidelines for the care and use of live fish at National Research Institute of Fisheries Science. First, an adult individual of Japanese eel, which was of full-life cycle culture (F2 generation derived from wild-caught grandparents) in National Research Institute of Aquaculture [42], was anesthetized with 0.2% 2-phenoxyethanol (Wako, Osaka, Japan) prior to sampling, and the genomic DNAs were then extracted from whole blood following the previously reported method [43]. Preparation of sequence templates for 454 FLX+ and Illumina GAII or HiSeq followed the manufacturers’ instructions. We first assembled 454 genomic shotgun and paired-end reads of 3 kb and 6 kb by Newbler assembler (version 2.9; Roche Diagnostics). Then the Illumina paired-end reads, mate-pair reads (75 or 100 bp × 2 with various insert lengths) and single reads (75 bp) spun off from quality screening of read pairs worked for improvement of sequence accuracy mapping them by bowtie2 (version 2.1.0) onto the scaffolds made up of 454 reads allowing one base indels [44]. As a result, 591,560 sites were overridden by sequences called by Illumina. The improved 454 scaffolds and contigs were then bridged by Illumina paired-end and mate-pair reads with SSPACE-basic (version 2.0) [45]. Bridging process was progressive in which paired-end (800 bp), mate-pair reads of 5 kb, 8 kb, 10 kb, and finally 15 kb worked sequentially (Additional file 1). Gaps in the scaffolds were filled by the Illumina reads with GapFiller (version 1.10) [46]. Total read coverage of the assembly was 129 × the expected genome size [47]. The Illumina paired-end reads (800 bp) were used also for genome size estimation based on *k*-mer frequency by JELLYFISH [48]. The scaffold sequences are deposited to DDBJ/GenBank/EMBL databases under accession numbers BDQN01000001–BDQN01195366. The linkage marker sequences obtained in the previous study [49] were mapped to the scaffold sequences using BLASTN (identity > = 90%). The markers matched to two or more scaffolds were excluded, and the scaffolds with single-hit markers were attributed to the linkage groups (LG1 to LG19). The correspondences to LGs were further manually checked, and the scaffolds attributed to two or more LGs were split into consistent ones based on the locations of marker. Scaffolds > = 2000 bp in length were used for subsequent analysis.

### RNA preparation and sequencing

From the same individual of Japanese eel as used for whole genome sequencing, brain, gill, esophagus, stomach, anterior intestine, posterior intestine, rectum, pancreas, liver, spleen, gall bladder, swim bladder, muscle, head kidney, body kidney, urinary bladder, blood, and skin were dissected out. All organs were immediately immersed in RNAlater stabilized solution (Thermo Fisher Scientific, Waltham, MA). Total RNA was extracted using the RNeasy Lipid Tissue Mini Kit (Qiagen GmbH, Hilden, Germany), according to the manufacturer’s protocol. RNA quality was evaluated based on the proportion of rRNA using an Agilent 2100 Bioanalyzer RNA 6000 Nano Kit (Agilent Technologies, Palo Alto, CA, USA). A complementally DNA libraries were constructed, followed by DSN Normalization using Duplex-Specific thermostable nuclease. Libraries were sequenced by Illumina HiSeq 2000 platform equipped with 100 bp paired-end module. After sequencing, the raw reads were filtered by removing low-quality reads (QV < 20).

### Gene prediction

The protein-coding genes in the Japanese eel genome were predicted using AUGUSTUS (version 3.2.2) [50]. First, RNA-seq reads of Japanese eel sequenced in this study were mapped to the scaffolds by TopHat [51] and assembled by Cufflinks [52]. In addition, protein sequences of 11 fish species: spotted gar (*Lepisosteus oculatus*), Mexican tetra (*Astyanax mexicanus*), zebrafish (*Danio rerio*), Atlantic cod (*Gadus morhua*), Nile tilapia (*Oreochromis niloticus*), platyfish (*Xiphophorus maculatus*), Amazon molly (*Poecilia formosa*), medaka (*Oryzias latipes*), stickleback (*Gasterosteus aculeatus*), greenpuffer (*Tetraodon nigroviridis*), and fugu (*Takifugu rubripes*), were downloaded from the ENSEMBL database (Release 84) [53], and mapped to the Japanese eel scaffolds by TBLASTN with E-value <10^−4^. Next, the scaffold sequences were scanned by the generic model in AUGUSTUS, using the map information of RNA-seq and ENSEMBL protein data as hints, and a total of 85,987 genes were predicted. Then, the predicted gene sequences were compared to the reference full-length protein sequences extracted from four fish genome data of ENSEMBL (zebrafish, medaka, greenpuffer, and fugu) by BLASTP [54] with E-value <10^−10^, and a total of 1776 sequences were selected as well-validated ones whose lengths were close to those of the reference sequences (difference < 5%). Using these sequences, the training model of eel genes was constructed, and the gene prediction was performed by AUGUSTUS again based on the above transcript and protein hints. Finally, InterProScan [55] was performed to the predicted gene sequences, and those matched by any domain or supported by any AUGUSTUS hint were collected as valid protein-coding genes.

### Synteny and phylogenetic analyses

The above-mentioned ENSEMBL data of 11 fish species were used for ortholog and synteny comparison. In addition, as outgroups in phylogenetic analysis, the gene sequences of chicken (*Gallus gallus*), and human (*Homo sapiens*) were also downloaded from the ENSEMBL. Furthermore, the genome data of two fish species, Asian arowana (*Scleropages formosus*) and northern pike (*Esox lucius*) [56], were downloaded from the GenBank (accession number: GCF_001624265) and the online resource at the University of Victoria (http://web.uvic.ca/grasp/pike/), respectively. From the transcriptome data of northern pike, protein-coding sequences were predicted by TransDecoder [57]. Orthologous genes among Japanese eel and these 15 species (13 fish species, chicken and human) were first estimated by OrthoMCL [58], and the genes missed by AUGUSTUS prediction or in the database annotation were further predicted by Exonerate [59] with protein identity >40% based on the protein sequences of Asian arowana and spotted gar. As a close relative of Japanese eel, the genome data of European eel (*Anguilla anguilla*) [60] was downloaded from the GenBank (accession number: GCA_000695075), and alignments of the scaffold sequences between these eels were constructed by MUMmer 3 [61].

For phylogenetic analysis, we selected species with good recovery of two syntenic regions in which Japanese eel retains *rho* paralogs (*rho* regions). Mexican tetra and platyfish were not included in further analysis because of disjuncture in these *rho* regions. Amino acid sequences were aligned with MAFFT ver. 6.7 [62] with manual adjustment. We pruned sites with gaps in more than a half of OTUs. DNA sequence alignments were deduced from the amino acid alignments. Nucleotides at fast evolving 3rd codon positions were treated in three ways for phylogenetic analysis: included in the analysis without modification, RY-coding [63] for accounting translations only, and excluded from the analysis. Substitution models were compared with MEGA ver. 6 [64]. We conducted phylogenetic analysis both on alignments of each separate gene sequences and on concatenated supermatrix sequences of the *rho* region. Maximum likelihood (ML) trees were inferred with PAML ver. 4.9 [65] by repeated local rearrangements [66]. Tree robustness was assessed by RELL method [67] with CONSEL ver. 0.2 [68]. Source of tree incongruence especially on position of the eel *fw-rho* was tested excluding sites around those showing higher non-synonymous substitutions detected with JCoda ver. 1.4 [69].

## Results

### Japanese eel genome and *rho* loci

We obtained a total of 20,564 scaffolds with 1055 Mb, accounting for 98.9% of the Japanese eel genome [47] (Table 1), while the genome size based on *k*-mer frequency was estimated to be 920 Mb (Additional file 2). Of these, 868 scaffolds totaling 529 Mb were attributed to each of 19 linkage groups of Japanese eel (Additional file 3). In 20,564 scaffolds, we predicted a total of 26,689 protein-coding genes, and from these detected two intronless rhodopsin genes, *ds-rho* and *fw-rho* (Additional file 4). These genes were located on two scaffolds, namely scaffold 3 (5,957,805 bp in total) on LG12 and scaffold 435 (438,394 bp in total) with no attribution to LG, respectively. In scaffold 3, a total of 127 genes including *ds-rho* were predicted. Since scaffold 435 was not fully assembled compared to scaffold 3, only eight genes including *fw-rho* were detected.

**Table 1:** Assembly statistics of the Japanese eel genome

No. of scaffolds	20,564		
Total base pairs (Mb)	1055		
Average scaffold size (kb)	51.3		
No. of scaffolds mapped to LGs	868		
Total base pairs mapped to LGs (Mb)	529		
Predicted protein-coding genes*	26,689		

*Predicted by AUGUSTUS and InterProScan.

**Fig. 2:**
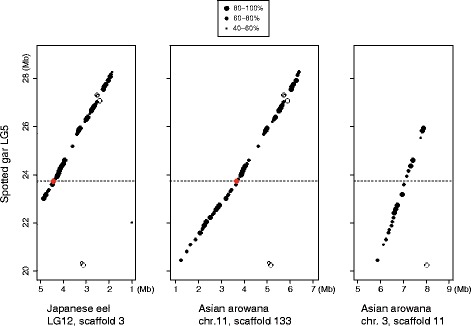
Dot plot for macrosynteny of *rho* region For Japanese eel scaffold 3, and Asian arowana scaffolds 133 and 11, the positions of genes matched to those in spotted gar’s LG5 region (20–29 Mb) are plotted. Each of the dots is the midpoint of predicted start and end positions of gene, and the size indicates the degree of amino acid identity (%) to the ortholog in spotted gar; in the same transcriptional direction (closed dot) or opposite direction (open dot). The rhodopsin gene is colored in red and the position in spotted gar’s LG5 is shown in dashed line.

Next, using these gene sequences in Japanese eel, we searched for orthologous genes in primitive ray-finned fish genomes, such as those of spotted gar and Asian arowana. We found that many of the genes in the Japanese eel scaffold 3 were orthologous to those encoded in LG5 of spotted gar. Particularly, the region of approximately 3 Mb surrounding *ds-rho* in Japanese eel had a large synteny to the *rho* region in LG5 of spotted gar (Fig. 2). Of 138 genes encoded in 20–29 Mb region of spotted gar’s LG5, 67 genes were detected in the *ds-rho* region of Japanese eel. The 57 genes were in the same transcriptional direction as those of spotted gar, and the other 10 genes were likely to be due to small inversions or translocations. We found that the Asian arowana genome also had two scaffolds syntenic to the *rho* region of spotted gar, namely scaffold 133 (6,725,481 bp in total) attributed to chromosome 11 and scaffold 11 (8,087,411 bp in total) attributed to chromosome 3, respectively (Fig. 2). In scaffold 133, 81 of 138 genes in spotted gar’s LG5 (20–29 Mb) were detected as orthologs, and 57 genes were common to those of the *ds-rho* region in Japanese eel. In scaffold 11, 28 of the spotted gar’s 138 genes were detected, but *rho* was absent.

**Fig. 3:**
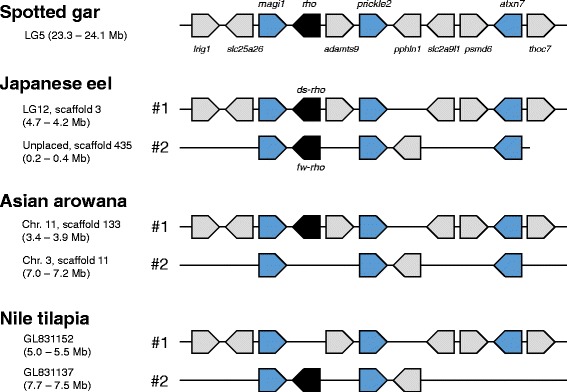
Microsynteny of 11 genes including *rho* Rhodopsin genes are colored in black, and three pairs of paralogs present in Japanese eel and Asian arowana, *magi1*, *prickle2* and *atxn7*, are highlighted in blue. In scaffold 435 of Japanese eel, *atxn7* was predicted at the end, and *thoc7* was not found in any other scaffolds. Putatively orthologous syntenies are denoted as #1 and #2, respectively.

The microsyntenies neighboring *rho* were further compared adding species of Otocephala (Mexican tetra and zebrafish) and Euteleostei (northern pike, Atlantic cod, Nile tilapia, platyfish, Amazon molly, medaka, stickleback, greenpuffer, and fugu). We focused on 11 genes including *rho* in the spotted gar LG5 as a reference, namely *lrig1*, *slc25a26*, *magi1*, *rho*, *adamts9*, *prickle2*, *pphln1*, *slc2a9l1*, *psmd6*, *atxn7*, and *thoc7* (Fig. 3). The order of 10 genes, except for *pphln1*, were perfectly conserved between Japanese eel’s scaffold 3 carrying *ds-rho* and spotted gar’s LG5. In addition, we found that the gene order in scaffold 133 of Asian arowana was identical to that in Japanese eel’s scaffold 3. Regarding *fw-rho* region, five genes (*magi1*, *rho*, *prickle2*, *pphln1*, and *atxn7*) were conserved between Japanese eel (scaffold 435) and spotted gar, but the other six were absent. To be precise, *atxn7* was predicted in the end of scaffold 435 and the absence of *thoc7* is unclear, but *thoc7* was not predicted in any other scaffolds. In scaffold 11 of Asian arowana, four (*magi1*, *prickle2*, *pphln1*, and *atxn7*) out of 11 genes were found. Therefore, except for absence of *rho*, this region of Asian arowana was syntenic to scaffold 435 of Japanese eel. For simplicity, we refer to these putatively orthologous syntenies of the *rho* region as #1 (eel scaffold 3 and arowana 133) and #2 (eel scaffold 435 and arowana 11). Regarding otocephalan and euteleostean species, the syntenic regions were also found in all the genomes examined, although the sequences were fragmented or some genes were not found (Fig. 3, and Additional files 5 and 6). All the cases were explainable by deletions of single gene in either of these syntenies. Pattern of the gene loss of *rho* paralogs was contrasting between Asian arowana and euteleosts examined.

Finally, we examined *rho* regions in the European eel genome. The scaffolds of European eel were more fragmented than those of Japanese eel, and the *ds-rho* region was split into two scaffolds in European eel (accession numbers: AZBK01S000177 and AZBK01S000303), but the gene order was eventually the same as Japanese eel (Additional file 7, Figs. S3a, b). Regarding *fw-rho* region, the scaffold of European eel (accession number: AZBK01S000274) was longer than that of Japanese eel (scaffold 435), whereby scaffold 435 was linked to a scaffold attributed to LG6 (scaffold 195,311) based on sequence alignment (Additional file 7, Fig. S3c).

### Phylogenetic analysis

**Fig. 4:**
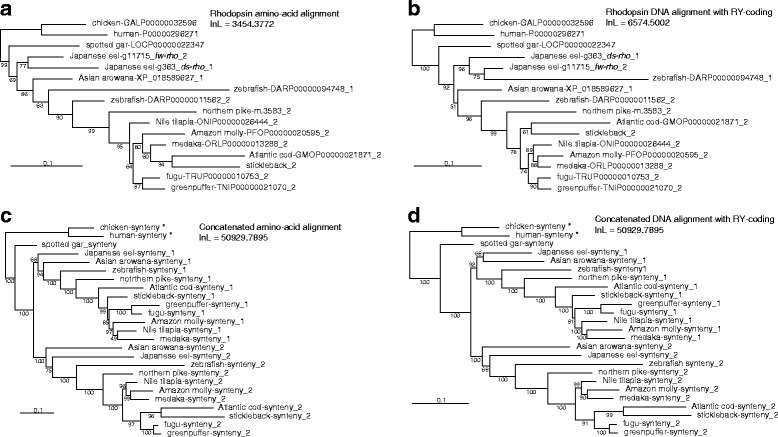
Phylogenetic trees Phylogenetic relationships among *rho* (**a**, **b**) and concatenated synteny (**c**, **d**) sequences. Amino acid (**a**, **c**) and RY-coded DNA (**b**, **d**) sequence-based trees are shown. Numbers attached to teleostean gene IDs denote syntenies on which genes are encoded. Numbers beside branches indicate local bootstrap [66] support (%) of each branch. * Chicken and human *rho* sequences of synteny data are from a remote locus.

Of eight genes commonly found in the *rho* region among vertebrates (Additional file 6), Japanese eel retains two copies of *magi1*, *rho*, *prickle2* and *atxn7* on two syntenies. We thus employed these four genes for phylogenetic analysis. Best fit models for phylogenetic analysis implemented in PAML were mostly JTT with gamma correction for amino acid alignments and GTR or TN93 with gamma correction for DNA alignments (Additional file 8). ML analysis of interrelationships among *rho* orthologs and paralogs gave skewed tree topologies (Figs. 4a, b, and Additional file 9). Two copies from Japanese eel got together in a clade independent from most of the others as was presented in previous studies [40, 70]. In addition, some disagreements in topology were observed among different sets of sequence alignment. Branch support values were low (81 and 84% on averages). Excluding sites around those with higher non-synonymous substitutions (Additional file 10) did not resolve this tree skewness (Additional file 9). Trees given on *atxn7* were also skewed (Additional file 9). Analysis of concatenated sequences, however, recovered two mutually monophyletic synteny clusters (Figs. 4c, d). Topologies given on different sequence datasets were mostly congruent with higher branch supports (both 93% on averages). Clades of Clupeocephala (zebrafish and higher teleosts) and Euteleostei (northern pike and higher) were recovered in each of two synteny clusters. Trees based on *magi1* and *prickle2* showed good recovery of monophyletic synteny clusters (Additional file 9).

## Discussion

We sequenced the genome of Japanese eel, mapped approximately half of the scaffolds assembled to linkage groups. Although the total size of scaffolds was highly consistent with the experimentally estimated genome size [47], it was ~15% larger than the estimate of genome size based on *k*-mer frequency. Since the genomes sizes of eel species have been recently reconsidered as with their repetitive regions and heterozygous sites [71], further improvements should also be done for the Japanese eel genome. In the present study, we focused on rhodopsin genes in Japanese eel and reconstructed the two loci of *rho* from the scaffold sequences. The *ds-rho* and *fw-rho* were located on different scaffolds, the longer one covering *ds-rho* was nearly 6 Mb in size, and the shorter one (~400 kb) had at least three paralogs (*magi1*, *prickle2* and *atxn7*) next to *fw-rho*. We can thus conclude that the duplication of *rho* in Japanese eel was neither at the single gene level nor tandem, as in the case of other visual pigment genes, but on a larger scale, and that it was followed by losses of neighboring paralogs. In addition, the scaffold of *ds-rho* widely corresponded to the region of spotted gar’s LG5, indicating that the genomic region surrounding *ds-rho* has kept the ancestral structure. Most importantly, we found that the Asian arowana genome had two scaffolds similar to those of *ds-rho* and *fw-rho* in Japanese eel, although a copy of *rho* was lost. Since the both scaffolds widely correspond to spotted gar’s LG5 region, the Asian arowana *rho* regions are products of a large scale duplication. Eel (Elopomorpha) and arowana (Osteoglossomorpha) are both considered to have been diverged at the early stage of teleost evolution [72]. This suggests a likely scenario for the origin of two regions of *rho* in Japanese eel and Asian arowana: a large scale duplication occurred in the common ancestor of eel and arowana, and after the divergence of these lineages, both *rho* genes have been maintained in Japanese eel, whereas a copy of *rho* has been lost in Asian arowana. An alternative scenario is that large scale duplications have occurred separately in each lineage after the divergences of eel and arowana, respectively, and a copy of *rho* has been lost in Asian arowana. Based on parsimony, this second scenario is less likely, because it assumes two parallel duplication events. Moreover, it should be noted that TSD is estimated to have occurred shortly before the divergences of eel and arowana [34, 73]. Thus, the second scenario needs further assumptions of loss of either of TSD-derived *rho* regions and recurrent parallel duplications. In the first scenario on the other hand, the observation can be simply explained by a single large-scale duplication before the divergences of eel and arowana and a loss of *rho* in the arowana lineage. Considering that the large scaffolds of Asian arowana were mapped on different chromosomes, it is reasonable to conclude that the large-scale duplication was TSD. In this study, we identified *ds-rho* of Japanese eel on the LG12 according to the mapped linkage markers. In addition, the comparison with the European eel genome suggested that *fw-rho* might be located on LG6. In the previous study, the LG12 and LG6 of Japanese eel or their backbone regions were estimated to have been built at TSD because of wide correspondences to spotted gar LG5 and medaka’s chromosomes 5 and 7 [49]. Thus, it seems likely that the *rho* genes in eel have been retained in the descendant regions of TSD-derived chromosomes.

**Fig. 5:**
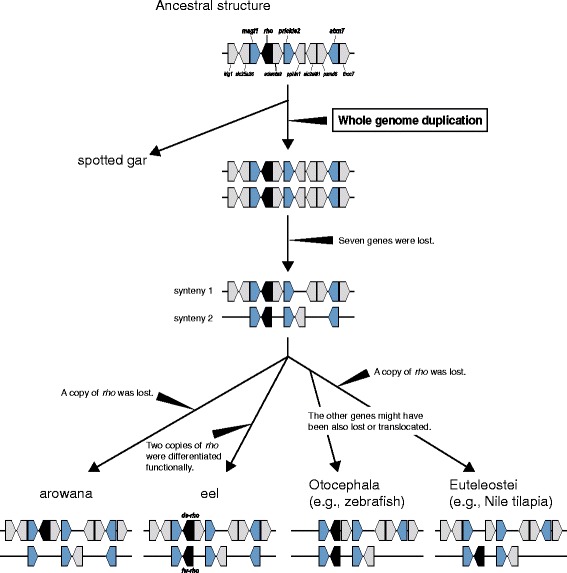
Evolutionary scheme of *rho* region in teleosts Rhodopsin genes are colored in black, and three pairs of paralogs present in Japanese eel and Asian arowana, *magi1*, *prickle2* and *atxn7*, are highlighted in blue.

Scaffold 435 of *fw-rho* in Japanese eel is short and its correspondence to Asian arowana’s scaffold 11 remains obscure, raising the additional possibility that *fw-rho* in Japanese eel has been generated by another duplication (e.g., lineage-specific segmental duplication). Although this third scenario also assumes the loss and a recurrent duplication of the *rho* region after TSD as in the second scenario, multiple losses and duplications may be possible on a smaller scale, such as the tandem duplications reported in other visual pigment genes. Therefore, we further examined the microsynteny around *rho* locus among Japanese eel and other teleosts. In spotted gar as an outgroup, *rho* was located within the region from *lrig1* to *thoc7* in LG5, and the synteny was also observed in the corresponding regions of Japanese eel and Asian arowana, strongly suggesting that the gene order is the ancestral form in teleosts. Considering that paralogs of *magi1*, *prickle2* and *atxn7* are present in the *fw-rho* region of Japanese eel, at least eight genes (*magi1*, *ds-rho*, *adamst9*, *prickle2*, *pphln1*, *slc2a9l1*, *psmd6*, and *atxn7*) as a template should have been doubled in the duplication event, followed by losses of several paralogs. Apparently, this is also the case in Asian arowana. Here, it is important to note that the pattern of gene losses was the same between Japanese eel and Asian arowana, except for that a copy of *rho* is lost in Asian arowana. Many studies have shown that either of the paralogs generated by duplication will be rapidly lost, while a number of paralogs might diverge functionally to each other [74–76]. The pattern of gene losses is a good signature for estimating the evolutionary scenario of duplicate genomic regions [77], because the genes lost once in the template region would never be restored by duplication again. In this study, for example, we can say that the *fw-rho* region with *pphln1* in Japanese eel should have been generated before the *pphln1* paralog was lost in the *ds-rho* region, and so too with those in Asian arowana. In particular, four genes, *adamst9*, *pphln1*, *slc2a9l1*, and *psmd6*, were lost in the same pattern between Japanese eel and Asian arowana. Assuming that the duplications occurred separately in each of eel and arowana lineages, these coincidences are unlikely. Moreover, in all the teleost genomes examined, we did not find any cases inconsistent with this pattern. Thus, these results suggest that the gene orders in two *rho* regions observed in Japanese eel had already emerged in the ancestor of eel, arowana, and clupeocephalan (Otocephala + Euteleostei) lineages (Fig. 5). Whereas zebrafish and Mexican tetra have two copies of *rho*, all the euteleosts examined have a single copy of *rho*, but the gene loss was in the opposite side from that in Asian arowana. This is also explained by our hypothesis that two *rho* genes were still present at the divergence of teleostean lineages. Thus, we propose that the losses of *rho* on opposite regions occurred independently in arowana and clupeocephalan lineages after their divergence; otherwise it would be difficult to explain the loss pattern of *rho* and *adamst9* paralogs between *magi1* and *prickle2*. Regarding two *rho* copies of zebrafish, phylogenetic analysis has recently suggested that the duplication occurred early in a teleost lineage, and either of the paralogs has evolved under positive selection [39]. According to our hypothesis, considering that the zebrafish lineage had branched at the base of Clupeocephala, it is possible that two *rho* copies predating the divergence of teleostean lineages have remained till the present in zebrafish and lost in the euteleostean ancestor. This possibility may be further tested by comparison to two *rho* copies in Mexican tetra in the same superorder (Otophysi) as zebrafish.

Two synteny clusters of the *rho* region among 11 teleosts in trees based on concatenated sequences (Figs. 4c, d) were in accord with the first scenario, because mutual monophyly of these synteny clusters can be traced back to the teleostean root. Recovery of outline of the teleostean interrelationships (clupeocephalan and euteleostean clades) in both synteny clusters with high branch supports indicates that the trees obtained from concatenated sequences are close to the true tree. The results from concatenated sequences, however, do not necessarily support the first scenario, because the two paralogous *rho* regions are merely vehicles of the gene of interest. Clustering of the eel *rho* copies relative to other teleosts in trees solely based on *rho* sequences regardless of exclusion of sites with higher non-synonymous substitutions (Figs. 4a, b and Additional File 10) is possible upon delayed resolution of tetrasomy [78] after TSD. Tetrasomy of chromosomal segments homogenizes homeolog sequences through recombination. Phylogenetic affinity of eel’s two *rho* copies could arise, if the *rho* region was tetrasomic and the resolution (evolution of paralogy) was delayed particularly in the eel lineage. *Atxn7*-based trees also show clusters of eel and/or arowana paralogs (Additional file 9). Allotetraploidy (no tetrasomy predicted) at TSD is suggested [79], but tetrasomy can occur even after allotetraploidization [80]. Another possibility for this skewed tree shape arises upon the second scenario with recurrent gene losses/duplications.

Nevertheless, dense taxon sampling of *rho* sequences [39], *magi1* and *prickle2* sequences in our study (Additional file 9) yielded similar results with those based on concatenated sequences. Differences of phylogenetic resolving powers in different sizes of sequence datasets (either or both dimensions of length and number of OTUs) would be a source of the incongruences. Our results suggest affinity of *ds-rho* and *fw-rho* with synteny 1 and 2 clusters respectively, and thus the origin of *ds-* and *fw-rho* genes can be traced back to the basal teleostean divergence near TSD. Thus, phylogenetic analysis supports the hypothesis that *rho* paralogs in Japanese eel have originated from TSD.

Finally, our results might shed light on the relationship between TSD and evolution of elopomorph species. Regarding sexual maturation, hormone receptor paralogs built at TSD, which are present in most other teleosts, have been examined also in eels [77, 81–83]. Moreover, genome-wide studies have shown that eels have the complete set of Hox gene clusters doubled at TSD [60, 84], while other teleosts lack some of the genes. Although such duplicate genes are likely involved in eels’ life cycle, the genes are largely conserved among teleosts and might not be directly correlated with eels’ unique adaptation to the freshwater and marine environments. Since the two rhodopsins in eel have been tuned for these environments, respectively [29, 36–38], our finding provides the first evidence about the functional differentiation of TSD-derived paralogs with special reference to the eel-specific ecological traits. The species which had deviated earlier in elopomorph divergence are distributed from estuary (e.g., tarpon) to deep sea (e.g., gissu). After their divergence, the lineages of eels and relatives (conger, gulper eel, etc.) are inferred to have diverged [73, 85, 86]. These studies imply that the aquatic habitats of elopomorph species have been often changed. Therefore, the present study raises the interest in the correlation between such a habitat alteration and the functions of rhodopsin in elopomorph species. For example, whether two copies of *rho* are maintained or either is lost in these species would be a simple but important topic. Japanese *Conger conger myriaster* has two copies of *rho* orthologous to those of Japanese eel, respectively [28], hence these genes are also derived from TSD as shown in this study. Previous studies showed functional differentiation of these *rho* copies in Japanese conger but in a different way from Japanese eel [28, 70, 87], suggesting multiple occurrence of the functional differentiation of *rho* paralogs by minor mutations in elopomorph fish retaining paralogs in their genome. In *Anguilla*, such as Japanese eel and European eel, the ancestral forms of *rho* may have been both deep-sea types, one of which has been mutated toward a freshwater-type, because a recent study suggested that these species evolved from a deep-sea habitat [88]. Thus, maintenance and sub-functionalization of the *rho* paralogs might be correlated with the wide distribution of elopomorph species from freshwater to the deep sea. It is difficult to answer in this study due to lack of other genome data on how often the functional changes of *rho* paralogs occurred in Elopomorpha. However, it should be stressed that maintenance of TSD-derived *rho* paralogs may have finally contributed to the establishment of eel’s life cycle, particularly the habitat alteration by long-distance migration between distinct aquatic environments. Further research based on genome-wide comparison may reveal whether or not this is a rare case with regard to the evolutionary impact of TSD.

## Conclusions

Two copies of eel rhodopsin gene were generated most likely at TSD. This is the first finding that TSD (~300 million years ago) has remotely lead to the gene innovation involved in eel-specific migratory life cycle. Further researches may provide insight into correlations between the functional differentiation of TSD-derived paralogs and the diversification of elopomorph species.

## Additional files

Additional file 1: Table S1. Sequencing results of the Japanese eel genome and cDNA. (XLS 32 kb)

Additional file 2: Figure S1. *K*-mer analysis using the Illumina paired-end reads of Japanese eel. (PPTX 42 kb)

Additional file 3: Table S2. LG mapping of the Japanese eel scaffolds. (XLS 335 kb)

Additional file 4: Table S3. Annotation of protein-coding genes predicted in the Japanese eel genome. (XLS 6125 kb)

Additional file 5: Figure S2. Microsynteny of *rho* region in other teleost genomes. (EPS 1575 kb)

Additional file 6: Table S4. Conservation of genes (*lrig1* - *thoc7*) around *rho* among vertebrates. (XLSX 15 kb)

Additional file 7: Figure S3. Comparison of *rho* regions between Japanese eel and European eel. (PDF 638 kb)

Additional file 8: Table S5. Substitution models employed in ML phylogenetic tree search. (XLS 28 kb)

Additional file 9: Supplement trees. (TXT 19 kb)

Additional file 10: Figure S4. Non-synonymous vs. synonymous substitution (dN/dS) ratio along *rho* sequence alignment with 25 aa window sliding every 5 aa. (PNG 70 kb)
