# Supplementary material for: Rhodopsin gene copies in Japanese eel originated in a teleost-specific genome duplication
Source: Zoological Lett. 2017 Oct 17;3:18. doi: 10.1186/s40851-017-0079-2 (PMC5645911; doi:10.1186/s40851-017-0079-2)
Supplement: Supplementary file 2 — K-mer analysis using the Illumina paired-end reads of Japanese eel. (PPTX 42 kb) [file 40851_2017_79_MOESM2_ESM.pptx]

## Slide 1
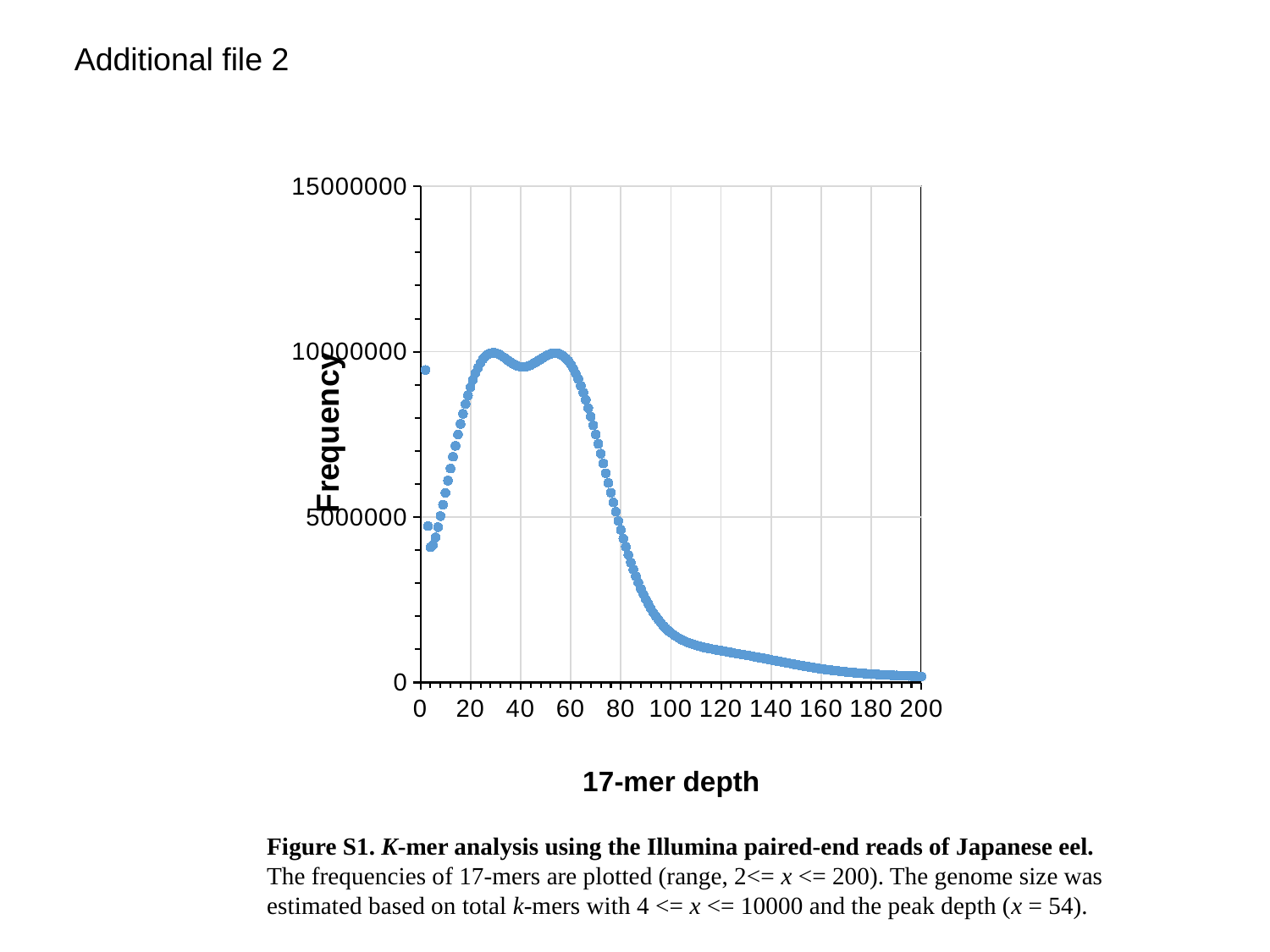

Additional file 2
### Chart
| Category | |
|---|---|Figure S1. K-mer analysis using the Illumina paired-end reads of Japanese eel.
The frequencies of 17-mers are plotted (range, 2<= x <= 200). The genome size was estimated based on total k-mers with 4 <= x <= 10000 and the peak depth (x = 54).
