# Supplementary material for: Rhodopsin gene copies in Japanese eel originated in a teleost-specific genome duplication
Source: Zoological Lett. 2017 Oct 17;3:18. doi: 10.1186/s40851-017-0079-2 (PMC5645911; doi:10.1186/s40851-017-0079-2)
Supplement: Supplementary file 7 — Comparison of rho regions between Japanese eel and European eel. (PDF 638 kb) [file 40851_2017_79_MOESM7_ESM.pdf]

Additional file 7

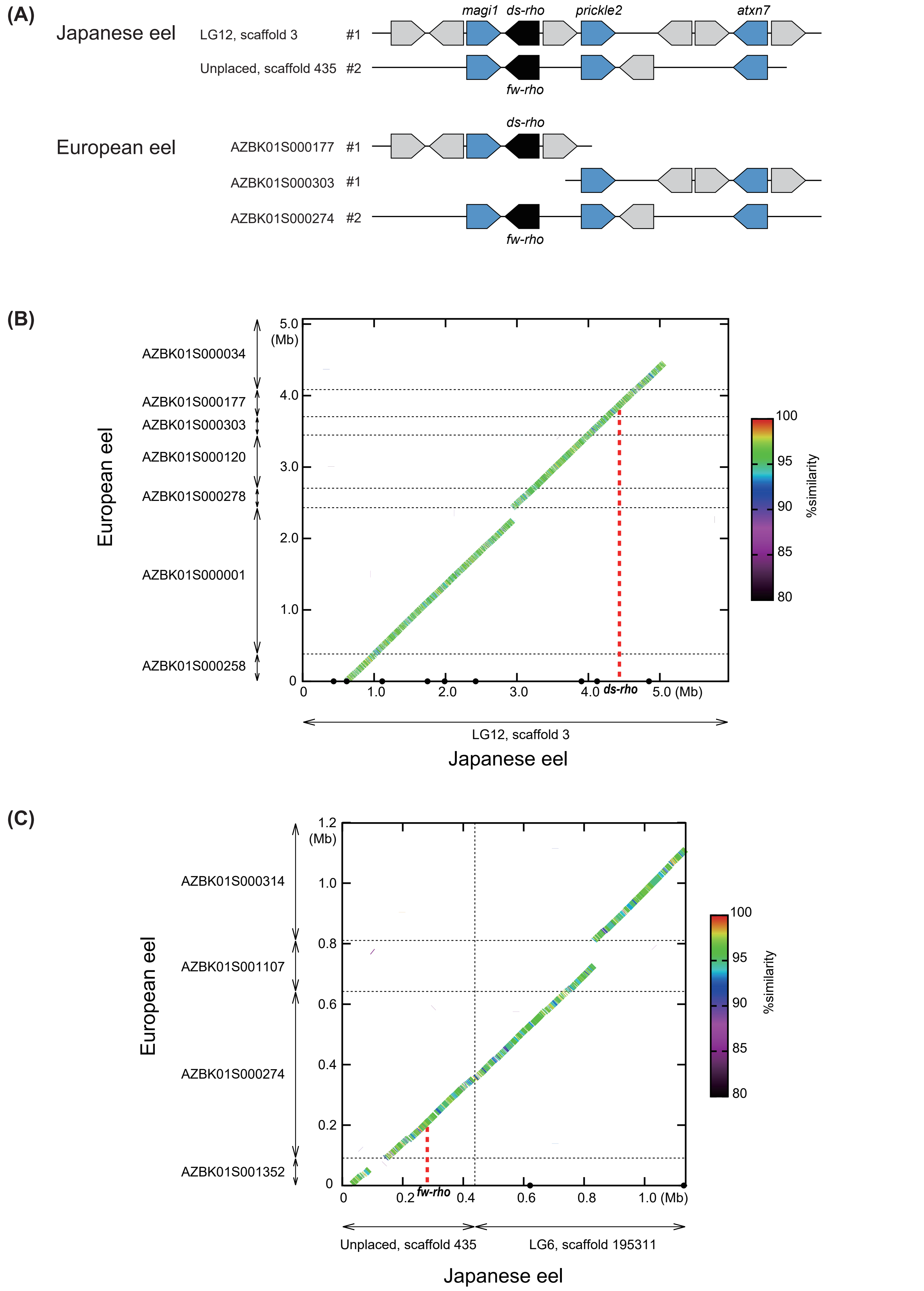

(continued on next page)

Figure S3. Comparison of *rho* regions between Japanese eel and European eel.

(A) Microsynteny of *rho* regions (*lrig1 – thoc7*). Numbering of syntenies (#1 and #2) and coloring of genes are according to those in Figure 3. (B and C) Comparison of scaffolds around *ds-rho* (B) and *fw-rho* (C) regions, respectively. The horizontal axis is for the scaffolds of Japanese eel and the vertical for those of European eel. The mapped positions of linkage marker in Japanese eel are plotted on horizontal axis.
