# Supplementary figures and images for: Rhodopsin gene copies in Japanese eel originated in a teleost-specific genome duplication
Source: Zoological Lett. 2017 Oct 17;3:18. doi: 10.1186/s40851-017-0079-2 (PMC5645911; doi:10.1186/s40851-017-0079-2)

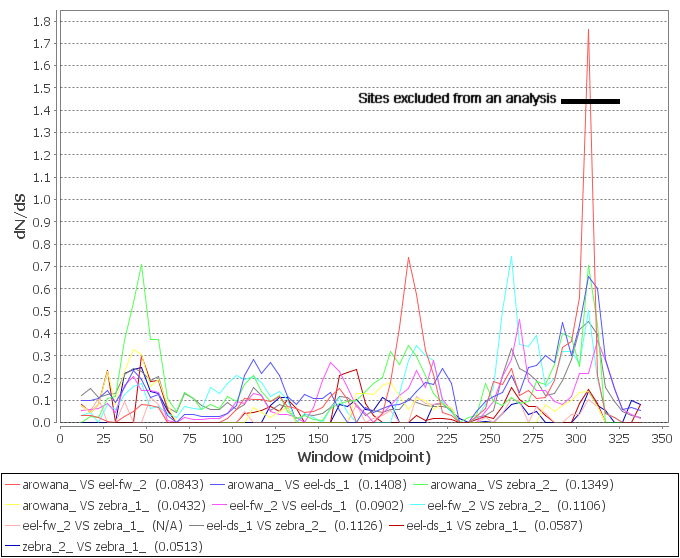

Supplement: Supplementary file 10 — Non-synonymous vs. synonymous substitution (dN/dS) ratio along rho sequence alignment with 25 aa window sliding every 5 aa. (PNG 70 kb) [file 40851_2017_79_MOESM10_ESM.png]
